# Workforce diversity among public healthcare workers in Nigeria: Implications on job satisfaction and organisational commitment

**DOI:** 10.1016/j.dib.2018.03.127

**Published:** 2018-03-31

**Authors:** Ayodotun Stephen Ibidunni, Hezekiah Olubusayo Falola, Oyebisi Mary Ibidunni, Odunayo Paul Salau, Maxwell Ayodele Olokundun, Taiye Tairat Borishade, Augusta Bosede Amaihian, Fred Peter

**Affiliations:** a Department of Business Management, Covenant University, Ota, Ogun State, Nigeria; b Department of Accounting, Bells University of Technology, Ota, Ogun State, Nigeria

**Keywords:** Workforce diversity, Job satisfaction, Employee commitment, Public healthcare, Diversity management

## Abstract

The aim of this research was to present a data article that identify the relationship between workforce diversity, job satisfaction and employee commitment among public healthcare workers in Nigeria. Copies of structured questionnaire were administered to 133 public healthcare workers from the Lagos state ministry of health in Nigeria. Using descriptive and structural equation modelling statistical analysis, the data revealed the relationship between workforce diversity and job satisfaction, workforce diversity and organisational commitment, and the role of job satisfaction on organisational commitment was also established.

**Specifications table**

**Table:** 

Subject area	*Strategic Management, Human Resource Management*
More specific subject area	*Organisational Planning, Diversity Management, Employee Satisfaction*
Type of data	*Table, figure*
How data was acquired	*Researcher made questionnaire analysis*
Data format	*Raw, analyzed, descriptive and statistical data*
Experimental factors	– *Samples consist of Public healthcare workers in Nigeria* – *In this paper, the perceptions of public healthcare workers about workforce diversity in relation to their satisfaction and commitment to the organisation was examined.*
Experimental features	*Healthy workforce diversity practices is a critical factor for sustaining employees’ job satisfaction and commitment*
Data source location	*Public Healthcare Workers from the Ministry of Health in Lagos State, Nigeria*
Data accessibility	*Data is included in this article*

**Value of the data**

• These data describe demographic data of public healthcare workers in the Nigerian health ministry, especially those in Lagos state.
• The data showed that workforce diversity, especially when it is based on gender and ethnicity, is very strategic to achieving employees' job satisfaction and commitment to their organisation.
• Moreover, the data is valuable to understanding the role of job satisfaction in enhancing their commitment to the organisation.
• The data from this study can be used to direct decisions that enhance meritorious distribution of power and benefits, based on workforce diversity factors to ensure employees job satisfaction and commitment.

## 1 Data

### 1.1 Demographic characteristics of respondents

Table 1 gives a distribution of gender of the respondents. 64 (48.1%) respondents are male while the remaining 69 (51.9%) respondents are female. This implies that there are more female respondents than the male respondents for this research.

**Table 1:** Gender distribution of respondents.

		Frequency	Percent	Valid percent	Cumulative percent
Valid	Male	64	48.1	48.1	48.1
	Female	69	51.9	51.9	100.0
	Total	133	100.0	100.0	

Table 2 shows age distribution of the respondents. 68 respondents are in the category of 18–34 years of age, 45 respondents fall within the range of 35–44 years of age. 13 respondents fall within the range of 45–54, and 7 respondents are in the category of 55 above years. This implies the age group which had the majority respondents was 18–34 years of age which had 68 respondents for this research.

**Table 2:** Age distribution of respondents.

		Frequency	Percent	Valid percent	Cumulative percent
Valid	18–34	68	51.1	51.1	51.1
	35–44	45	33.8	33.8	85.0
	45–54	13	9.8	9.8	94.7
	55 and above	7	5.3	5.3	100.0
	Total	133	100.0	100.0	

Table 3 classifies the respondents by the income received. The table reflects that (6.8%) of the respondents received an income range of 18,000 and below, (8.3%) of the respondents received an income of 18,001–37,000, (11.3%) of the respondents received an income of 37,001– 44,000, (28.6%) of the respondent received an income of 44,001– 71,000, (18.8%) of the respondent received an income of 71,001– 145,000, and (28.3%) of the respondent received an income of 145,001 and above. This implies the income range group which had majority respondents was 145,001 and above which had 35 respondents for this research.

**Table 3:** Income status of respondents.

		Frequency	Percent	Valid percent	Cumulative percent
Valid	below 18,000	9	6.8	6.8	6.8
	18,001–37,000	11	8.3	8.3	15.0
	37,001–44,000	15	11.3	11.3	26.3
	44,001–71,000	38	28.6	28.6	54.9
	71,001–145,000	25	18.8	18.8	73.7
	145,001 above	35	26.3	26.3	100.0
	Total	133	100.0	100.0	

Table 4 shows educational background of the respondents in this research 24 (18.0%) attained O.N.D, 9 (6.8%) respondents had N.C.E, while 43 (32.3%) attained H.N.D, 35 (26.3%) attained Bachelor's degree, and 22 (16.5%) had master’s degree. This implies that the majority educational background was (32.3%) which implies that 43 respondents attained H.N.D.

**Table 4:** Educational qualification of respondents.

		Frequency	Percent	Valid percent	Cumulative percent
Valid	O.N.D	24	18.0	18.0	18.0
	N.C.E	9	6.8	6.8	24.8
	H.N.D	43	32.3	32.3	57.1
	Bachelor's degree	35	26.3	26.3	83.5
	Master's degree	22	16.5	16.5	100.0
	Total	133	100.0	100.0	

Table 5 shows the ethnicity of the respondents in this research. The table shows that 81 (60.9%) of the respondents are Yoruba, 7 (5.3%) of the respondents are Hausa/Fulani, 30 (22.6%) of the respondents are Igbo and 15 (11.3%) of the respondents are from other tribes. This implies that the majority of Ethnicity was (60.9%) which implies that 81 respondents are Yoruba.

**Table 5:** Ethnicity of respondents.

		Frequency	Percent	Valid percent	Cumulative percent
Valid	Yoruba	81	60.9	60.9	60.9
	Hausa/Fulani	7	5.3	5.3	66.2
	Igbo	30	22.6	22.6	88.7
	Other tribe	15	11.3	11.3	100.0
	Total	133	100.0	100.0	

Table 6 classifies the respondents by years of work experience. The table reflects that (40.6%) of the respondents had worked for less than 5 years, the table reflects that (36.8%) of the respondents had worked for 5–10 years, the table reflects that (12.0%) of the respondents had worked for 11–15 years and the table reflects that (10.5%) of the respondents had worked for 16 years above.

**Table 6:** Years of work experience of respondents.

		Frequency	Percent	Valid percent	Cumulative percent
Valid	Less than 5 years	54	40.6	40.6	40.6
	5–10 years	49	36.8	36.8	77.4
	11–15 years	16	12.0	12.0	89.5
	16 years above	14	10.5	10.5	100.0
	Total	133	100.0	100.0	

Table 7 classifies the respondents by organisational status. The table reflects that (40.6%) of the respondents are junior level staff, the majority of the respondents are (45.1%) middle level staff and the table reflects that (14.3%) of the respondents are top (management) staff.

**Table 7:** Organisational status of respondents.

		Frequency	Percent	Valid percent	Cumulative percent
Valid	Junior level staff	54	40.6	40.6	40.6
	Middle level staff	60	45.1	45.1	85.7
	Top (management) staff	19	14.3	14.3	100.0
	Total	133	100.0	100.0	

Table 8 shows the distribution of religion/belief of respondents. 2 (1.5%) respondents have no religion or belief/ Atheist, majority of respondents were Christians 103(77.4%) and 28 (21.1%) are Muslims.

**Table 8:** Religion/belief of respondents.

		Frequency	Percent	Valid percent	Cumulative percent
Valid	No religion or belief/Atheist	2	1.5	1.5	1.5
	Christianity	103	77.4	77.4	78.9
	Muslim	28	21.1	21.1	100.0
	Total	133	100.0	100.0	

Table 9 classifies the respondents by marital status. The table reflects that 43 (32.3%) of the respondents were single. The majority of the respondents were married 87 (65.4%), the table reflects that 1 (0.8%) of the respondents was divorced/separated while 2 (1.5%) of the respondents were widowed (Table 10).

**Table 9:** Marital status of respondents.

		Frequency	Percent	Valid percent	Cumulative percent
Valid	Single	43	32.3	32.3	32.3
	Married	87	65.4	65.4	97.7
	Divorced/ separated	1	0.8	0.8	98.5
	Widowed	2	1.5	1.5	100.0
	Total	133	100.0	100.0

**Table 10:** Structural regression weights for workforce diversity, job satisfaction and employee commitment.

Variables			Estimate	S.E.	C.R.	*P*	Decision
JobSatisf	<---	Gender	0.392	0.093	4.224	***	H_0_ Reject
JobSatisf	<---	Education	0.055	0.066	0.845	0.398	H_0_ Accept
JobSatisf	<---	Ethnicity	0.392	0.111	3.530	***	H_0_ Reject
JobSatisf	<---	Position	0.066	0.091	0.719	0.472	H_0_ Accept
AffectCom	<---	Gender	0.157	0.065	2.405	0.016	H_0_ Accept
NormCom	<---	Gender	0.237	0.068	3.480	***	H_0_ Reject
ContCom	<---	Gender	0.105	0.089	1.184	0.237	H_0_ Accept
AffectCom	<---	Education	0.057	0.043	1.332	0.183	H_0_ Accept
NormCom	<---	Education	0.093	0.047	1.988	0.047	H_0_ Accept
ContCom	<---	Education	0.092	0.057	1.609	0.108	H_0_ Accept
AffectCom	<---	Religion	0.083	0.052	1.586	0.113	H_0_ Accept
NormCom	<---	Religion	0.065	0.057	1.148	0.251	H_0_ Accept
ContCom	<---	Religion	0.124	0.069	1.788	0.074	H_0_ Accept
ContCom	<---	Ethnicity	−0.074	0.092	−0.803	0.422	H_0_ Accept
AffectCom	<---	Experience	0.042	0.052	0.802	0.423	H_0_ Accept
NormCom	<---	Experience	−0.008	0.047	−0.170	0.865	H_0_ Accept
ContCom	<---	Experience	0.140	0.066	2.113	0.035	H_0_ Accept
AffectCom	<---	Income	−0.054	0.034	−1.607	0.108	H_0_ Accept
ContCom	<---	Income	−0.043	0.041	−1.064	0.287	H_0_ Accept
AffectCom	<---	Position	0.011	0.075	0.152	0.879	H_0_ Accept
ContCom	<---	Position	0.040	0.089	0.445	0.656	H_0_ Accept
AffectCom	<---	JobSatisf	0.273	0.055	4.977	***	H_0_ Reject
NormCom	<---	JobSatisf	0.185	0.059	3.116	0.002	H_0_ Accept
ContCom	<---	JobSatisf	0.042	0.075	0.565	0.572	H_0_ Accept

^***^ indicates that there is a significant relationship between the predictor variable and the corresponding explanatory variable.

**Fig. 1:**
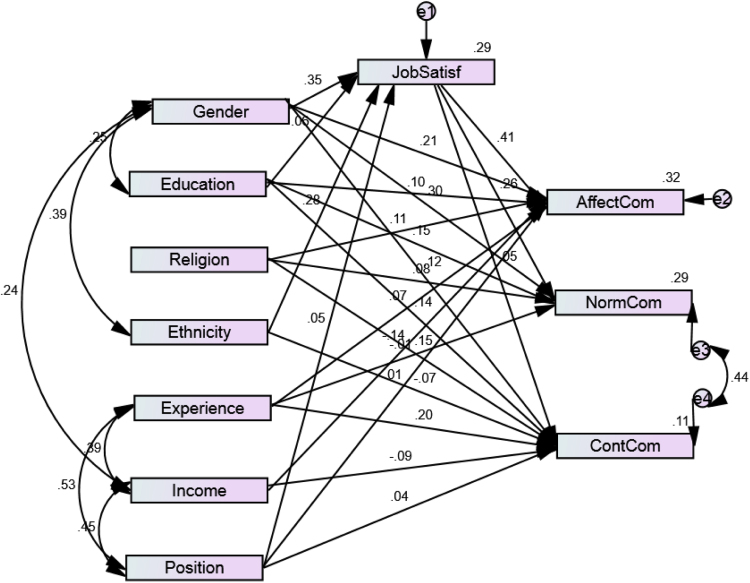
Path analysis of workforce diversity, job satisfaction and employee commitment.

Fig. 1 shows the path analysis model of multivariate relationships between workforce diversity, job satisfaction and employee commitment. Table 2 also shows the structural regression weights of the multivariate analysis. The model fit of the analysis is assured by the following indicators Chi-square/Degree of Freedom (Cmin/df)=1.603, Goodness of Fit Index (GFI)=0.950, Normed Fit Index (NFI)=0.892, Comparative Fit Index (CFI)=0.952, Root Mean Square Error of Approximation (RMSEA)=0.068. The values are significant based on the arguments presented in established studies [9], [10], [11].

The data supports a relationship between workforce diversity and job satisfaction, workforce diversity and organisational commitment, and the influence of job satisfaction on organisational commitment was also established. The findings from this data supports the outcomes from existing research, for example [8], [12], [13], [14], [15].

## 2 Experimental design, materials and methods

Survey method was adopted to gather data. 133 public healthcare workers from the Lagos state ministry of health in Nigeria were included in the research. Public healthcare workers, particularly in Nigeria's ministry of health are significant to this research because of the increasing awareness that relates to diversified workforce within the ministry and the need to manage such diversity in a way that will not have negative effect on employees' job satisfaction and their commitment to the organisation [1]. More so, because of the importance of the ministry of health to the populace and overall wellbeing of any nation, this research is considered pivotal, as a means of sustaining employees' interest and motivation to quality service delivery [2], [16]. Questionnaire was used to gather primary data from the respondents. This research benefitted from the ideas of existing research studies. Questions that pertained to workforce diversity was developed based on [3], [4], items for job satisfaction was developed based on [5], [6], while items for employee commitment was developed based on [7], [8]. The collated data were coded and entered in SPSS version 22. Data analysis was performed applying descriptive statistics and structural equation modelling (SEM). Ethical consideration in the research process was ensured because administering the questionnaires to respondents was based on their willingness to respond to the research instrument. Moreover, confidentiality and anonymity for participants in the study was assured.

## 3 Conclusion and implications of the study

The data presented reveals that workforce diversity significantly influences job satisfaction and commitment among public healthcare workers in Nigeria. The data has significant implications in directing the efforts policy makers towards ensuring a balanced mixed of demographic diversity among employees in the public health sector. The study serve as guide in the recruitment process of workers in this sector. More so, the data presented in this article is significant to guiding further investigations in extensive research.
