# Supplementary material for: Workforce diversity among public healthcare workers in Nigeria: Implications on job satisfaction and organisational commitment
Source: Data Brief. 2018 Mar 31;18:1047–53. doi: 10.1016/j.dib.2018.03.127 (PMC5996598; doi:10.1016/j.dib.2018.03.127)
Supplement: Supplementary file 1 — Supplementary material [file mmc1.doc]

Ibidunni, Ayodotun Stephen (PhD)

Covenant University,

Ota,

Ogun State,

Nigeria

21st March, 2018

The Editor,

Data In Brief,

Dear Sir,

**DECLARATION OF CONFLICT OF INTEREST**

I, Dr. Ibidunni, Ayodotun Stephen and my colleagues write to declare that there is no conflict of interest traceable to our data paper “***Workforce Diversity among Public Healthcare Workers in Nigeria: Implications on Job Satisfaction and Organisational Commitment***”

Yours faithfully,


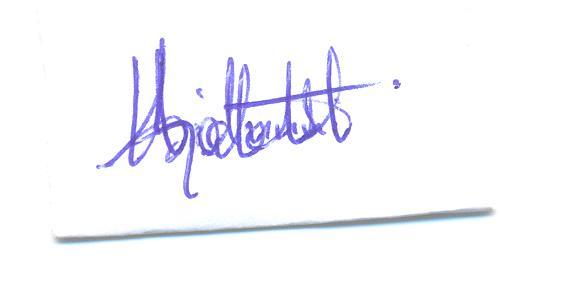


Ibidunni, Ayodotun Stephen (PhD) (Corresponding Author)

+234-803-489-3637
